# The Fitting of a Fiber-Reinforced-Plastic Complex Curved Surface and Its Orbit Optimization Model with Belt Grinding Line Contact

**DOI:** 10.3390/ma12172688

**Published:** 2019-08-22

**Authors:** Jiazheng Xing, Guijian Xiao, Yi He, Yun Huang, Shuai Liu

**Affiliations:** The State Key Laboratory of Mechanical Transmissions, Chongqing University, No.174, Shazhengjie, Shapingba, Chongqing 400044, China

**Keywords:** FRP complex curved surface, belt grinding line contact, least-squares fitting, trajectory optimization

## Abstract

The surface quality and profile accuracy of a radar fiberglass radome are determined by the manufacturing of the fiber-reinforced-plastic (FRP) complex curved mold. The surface quality, thickness uniformity, and shape accuracy of the mold seriously affect the temperature and deformation control during the manufacturing process of the radome, thus affecting the antenna’s serviceability, including its wave permeability and stability. Abrasive belt grinding is an effective method for processing FRP materials. However, issues regarding the profile fitting of the abrasive belt section line contact state and its influence on the precision of complex curved surfaces have not been solved, which seriously affects the processing quality. Here, an FRP complex curved surface mold surface based on the least-squares method was established. The local two-dimensional line contact and profile contour trajectory were obtained by the algorithm of optimal trajectory planning. Based on this, a grinding experiment was carried out. The experiments showed that the surface roughness based on this method was reduced from 0.503 to 0.289 μm, and the contour accuracy was improved by 16.9% compared with the conventional error. Through our analysis, the following conclusions can be drawn: the algorithm can effectively solve the problem of line contact surface fitting and significantly improve the precision of an FRP complex surface.

## 1. Introduction

The surface quality and profile accuracy of a radar fiberglass radome are determined by the manufacturing of the fiber-reinforced-plastic (FRP) complex curved mold. The surface quality, thickness uniformity, and shape accuracy of the mold seriously affect the temperature and deformation control during the manufacturing process of the radome, thus affecting the serviceability of the antenna, including its wave permeability and stability. Therefore, it is of great significance to improve the surface quality and profile accuracy of FRP complex curved abrasive tools [1].

As FRPs have the characteristics of high cutting heat, high fiber hardness, easy removal, easy burning, and heterogeneous structure, the cutting process is a major difficulty. Relatively speaking, the grinding process is more suitable for processing FRPs than the abovementioned processing methods, but it also poses many problems [2,3]. FRPs are very dusty during grinding, which can cause operational difficulties in addition to being harmful to human health. The grinding heat generated during the FRP grinding process is very strong and can result in severe burns on the grinding surface and increased wear on the grinding wheel. Since the adhesion on the grinding wheel is very strong, it is not easily cleaned and requires timely repair or even replacement of the grinding wheel, which greatly increases costs [4,5,6,7,8]. Abrasive belt grinding is an economical, efficient, and versatile new grinding process referred to as “universal grinding”. In addition to the grinding effect on the machined surface, it also has the effect of polishing. The advantages of abrasive belt grinding are that there is almost no heat on the surface during grinding, the surface integrity is good after grinding, and the surface roughness is low [9,10]. However, issues regarding the profile fitting of the abrasive belt section line contact state and its influence on the precision of complex surfaces have not been solved, which seriously affects the processing quality [11].

As for the material processing problems for FRPs, machining processes such as turning, milling, planning, grinding, drilling, riveting, and reaming are mainly used in China. Of course, in addition to these common processing methods, there are special processing methods also used in China, such as laser processing, high-pressure water jet processing, and ultrasonic processing. Although these technologies have relatively high processing efficiencies, these methods also have many problems due to technical defects, such as the accuracy not being up to the expected value and the process being harmful to human health. Several countries have been leading the world in the development of FRPs for decades [12,13]. Therefore, their research on FRP processing is advanced. To address the problems of low FRP processing efficiency and defects such as delamination being easily generated during processing, Won predrilled a work piece to reduce the effect of the twisted edge of the twist drill, thus obtaining the twist drill. They examined the influence of the chisel edge on the axial force of the cutting and further derived the critical axial force model of the predrilled hole based on the theoretical model of critical axial force obtained by Hocheng. They proposed and verified the following conclusions: when the twist drill is used for FRP drilling, if the axial force of the drilling is less than the critical axial force, then the higher the feed speed, the higher the processing efficiency of the FRP, and defects such as delamination can be effectively avoided [14,15,16].

For the modeling of FRP contour surfaces, Wang et al. used high-dimensional data points to enrich the hull surface value information and developed a 3D modeling process of an FRP hull free-form surface by using a database which rapidly classified the value point data, thus greatly improving the design efficiency and accuracy of an FRP boat. However, this method is relatively inefficient for hood profile surfaces [17]. Oqielat proposed a new surface fitting method based on a fused Gaussian radial basis function (RBF) and carat tolerance (CT) called RBF-CT. Applying this method to the scattering data of Franke (1982) and the real scattering dataset collected by laser scanner of *Anthurium* leaves (Loch, 2004), the accuracy of the method was verified and a foliate surface was constructed for this. However, this method does not apply to the modeling of FRP contour surfaces [18].

Surface prediction is especially important for the complex surface of a glass radome before polishing. The surface prediction accuracy determines the base value of systematic error during the grinding phase. For the prediction of the curve, the methods of interpolation or fitting can be used. For this problem, there are higher requirements for the overall prediction effect, and abnormal prediction values are not allowed. Therefore, in this study, we chose to use the fitting method. Although there are problems regarding the processing of FRP materials and the modeling of FRP contour surfaces, no surface modeling and error analysis has been developed for the abrasive belt contact state. To address this, we have proposed a contact fit trajectory optimization model of an FRP complex curved belt grinding line. An FRP complex surface mold surface based on the least-squares method was established. The local two-dimensional line contact and profile contour trajectory were obtained by the algorithm of optimal trajectory planning. Based on this, an error analysis was carried out on an experimental platform, and experimental verification was carried out.

## 2. Glass Radome Complex Surface Mathematical Experiment

### 2.1. Modeling and Error Analysis of FRP Complex Surface Based on Least-Squares Method

There are many methods of fitting. In addition to high-accuracy prediction of the whole problem, there is also a very high requirement for the maximum local deviation of the prediction accuracy. In order to guarantee the base value of the systematic error in the grinding stage, the maximum local deviation must be below 1%. Therefore, the least-squares method was selected. The least-squares method finds the best match of the data by minimizing the square of the deviation, which ensures the control of the overall prediction. Secondly, it is very sensitive to outliers and controls the local deviation. This ensures that the local deviation is not too great.

In this problem, since the glass radome is fully symmetrical about the central axis, a cross section was made through the central axis, and the complex curved surface of the entire radome was predicted by predicting the function of the profile. To this end, the model of the radome was converted from digital to analog, and the relative coordinates of 46 discrete points on a section were obtained. As shown in Table 1.

The basic idea of the linear least-squares method is as follows:

(1) $$\begin{array}{c} f\left(x\right)=a_{1}r_{1}\left(x\right)+a_{2}r_{2}\left(x\right)+a_{3}r_{3}\left(x\right)+\cdots a_{m}r_{m}\left(x\right). \end{array}$$

This is a set of linearly independent functions selected in advance, which are pending coefficients (*k = 1, 2,* ⋯*, m*, *m* < *n*). The fitting criterion is to make $y_{i}\;\left(i=\;1,\;2,\;\cdots ,\;46\right)$, and the sum of the squares of the distances $\delta_{i}$ from $f\left(x_{i}\right)$ is the smallest.

To determine the coefficients, a cost function is constructed:

(2) $$J\left(a_{1},\cdots ,a_{m}\right)=\overset{n}{\underset{i=1}{\sum }}\delta_{i}{}^{2}=\overset{46}{\underset{i=1}{\sum }}{\left[f\left(x_{i}\right)-y_{i}\right]}^{2}.$$

In order to minimize the cost function *J*, it is only necessary to use the extremely necessary conditions $\frac{\partial J}{\partial a_{k}}=0\;\left(k=1,\;\cdots ,m\right)$ to obtain the linear equations $a_{1},\;\cdots ,a_{m}$ and solve for the unique solution.

For the selection of the function $r_{k}\left(x\right)$, the epitaxial curve of the radome has excellent curved surface features, while the function with better curved features has a polynomial function and a trigonometric function. Therefore, we considered fitting with polynomial and Fourier approximations. Combined with the grinding requirements of the glass radome, not only are the global fitting requirements high, but the local maximum deviation is also below 1%. For this reason, three indicators were used to evaluate the pros and cons of the fitting.

*SSE* is a statistical parameter that calculates the sum of the squares of the errors of the fitting data and the corresponding points of the original data. The closer the SSE is to 0, the better the model selection and fitting. This indicator is used to measure the global fit.

*R*^2^ (determination coefficient):

(3) $$\begin{array}{c} SSE\left(Intragroup\;variation\right)+SSA\left(Inter-group\;variation\right)=SST\left(Total\;variation\right)\\ R-square=\frac{SSR}{SST}=\frac{SST-SSE}{SST}=1-\frac{SSE}{SST}\;, \end{array}$$

where *SSR* is the sum of the squares of the difference between the predicted data and the mean of the original data, and *SST* is the sum of the squares of the difference between the original data and its average. The determination coefficient is a change in the data to characterize the fit of a fit. The closer it is to 1, the stronger the ability of the equation to interpret *y*, and the model fits the data better.

C (maximum relative deviation):

(4) $$\begin{array}{c} C=\max\left(\frac{f\left(x_{i}\right)-y_{i}}{y_{i}}\right)*100\%\;i\;=\;1,\;2,\;\ldots ,\;n. \end{array}$$

**Figure 1:**
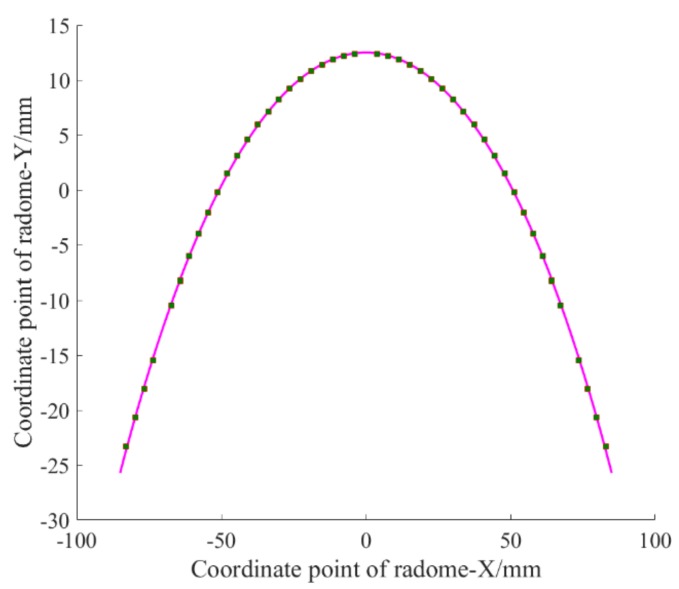
Fitting function with polynomial approximation.

This is used to measure the local maximum deviation. To do this, we first used a polynomial approximation, and the fitting graph is shown in Figure 1.

The fitting function expression is $f\left(x\right)=-0.004616x^{2}+12.51$.

The fitting effect is *SSE* = 0.7634, *R*^2^ = 0.9999, and C = 2.18%.

The advantage of this fitting result is that the fitting function is simpler in form and the overall fitting effect is better, but there are individual “singularities” (that is, the local maximum deviation that does not reach our expectation, where local deviation below 1% is our expectation).

Since the fitting function has better characteristics, it satisfies the form of $F\left(x\right)=\overset{m}{\underset{i=1}{\sum }}f_{i}{}^{2}\left(x\right),x\epsilon R^{n}x={\left(x_{1},\cdots ,x_{n}\right)}^{T}$. Therefore, it is also possible to classify such problems as least-squares optimization problems, which minimize the function

(5) $$\begin{array}{c} \min F\left(x\right)=\sum_{i=1}^{m}f_{i}{}^{2}\left(x\right). \end{array}$$

**Figure 2:**
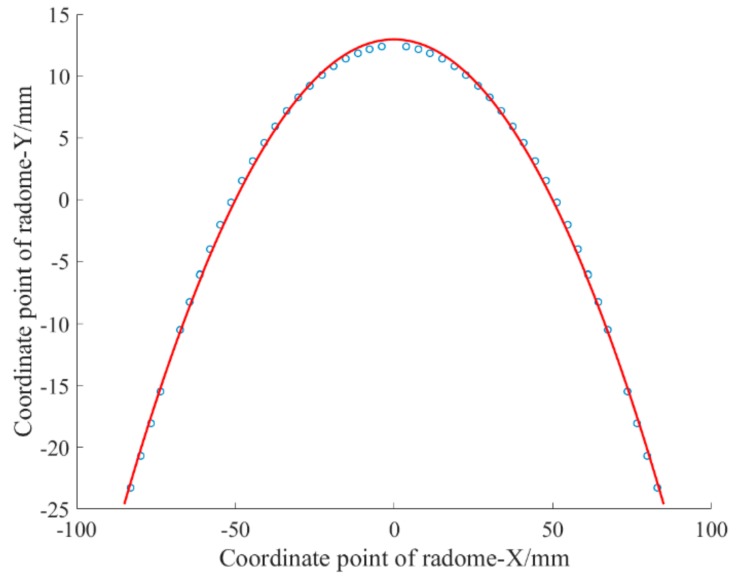
Least-squares optimization fit curve.

As the target equation, the fitting curve is also obtained from it (Figure 2).

The fitting function expression is $\left(x\right)=-0.0052x^{2}+12.675$.

The fitting effect is *SSE* = 1.5432, *R*^2^ = 0.9988, and C = 8.32%.

The overall fitting effect of this function is not as good as the former. For this reason, although polynomial fitting can obtain a relatively simple function expression and the overall fitting effect is better, there are some singularities (the local maximum deviation is higher than 1%). Therefore, when grinding a glass radome that is not very high precision, the polynomial fitting result can be used as a reference function of the numerical control machine program. The advantage is that the function expression is extremely simple.

**Figure 3:**
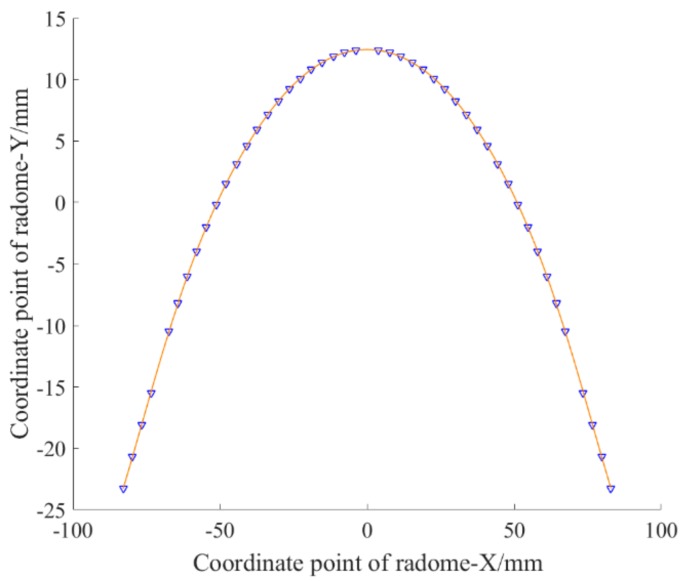
Fourier approximation fitting curve.

In order to avoid the occurrence of singularity, the simplicity of the function can only be sacrificed to meet the demand, and the Fourier approximation form was adopted for this purpose. The fitting graph is shown in Figure 3.

The fitting function expression is:

$$\begin{array}{ll} f\left(x\right) & =177.3\sin\left(\mathrm{wx}\right)-260\sin\left(2\mathrm{wx}\right)+229.8\sin\left(3\mathrm{wx}\right)-142.3\sin\left(4\mathrm{wx}\right)+63.02\sin\left(5\mathrm{wx}\right)\\ & -19.28\sin\left(6\mathrm{wx}\right)+3.692\sin\left(7\mathrm{wx}\right)-2190\cos\left(8\mathrm{wx}\right)-0.3364\sin\left(8\mathrm{wx}\right)w=0.01164. \end{array}$$

The fitting effect is *SSE* = 0.0098, *R*^2^ = 1.000, and C = 0.6%.

For this problem, it is obvious that the fitting effect of the Fourier approximation is excellent. Whether it is the whole or the part, the control of the precision is in place, and there is no “bad point” which is perfect. The only shortcoming is that its function expression is more complicated, and it is more troublesome in NC programming.

**Figure 4:**
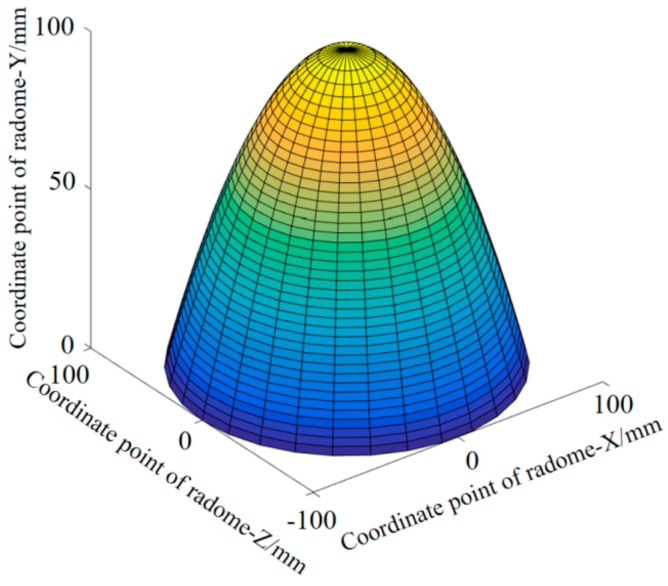
Three-dimensional model diagram of the Fourier approximation fitting function.

In order to more intuitively reflect the fitting effect of the Fourier approximation, the fitting function was applied to the overall outer surface prediction of the radome. Since the radome has central axis symmetry, the fitting curve was rotated around the *y*-axis, as shown in Figure 4. 

The prediction accuracy of the Fourier approximation function is visually reflected in the form of an error “cloud map”. Since the absolute error points held are discrete points, the inverse distance weighting (IDW) algorithm was adopted to reflect the overall accuracy error and three-dimensional interpolation was performed.

Calculate the z-value of any point on the plane using the known *i* points:

(6) $$\;z=\overset{n}{\underset{i=1}{\sum }}w_{i}z_{i}$$

(7) wi=1diq∑i=1n1diq.

The weights are

(8) $$\begin{array}{c} d_{i}={\left[{\left(x_{1}-x_{i1}\right)}^{2}+{\left(x_{2}-x_{i2}\right)}^{2}+\cdots +{\left(x_{n}-x_{in}\right)}^{2}\right]}^{\frac{1}{n}}.\; \end{array}$$

When *q* is larger, the closer the distance of $\left(x_{i},y_{i}\right)$ between $\left(x_{n},y_{n}\right)$, the greater and the farther the relative effect. In this problem, *q* = 3 has a better interpolation effect.

**Figure 5:**
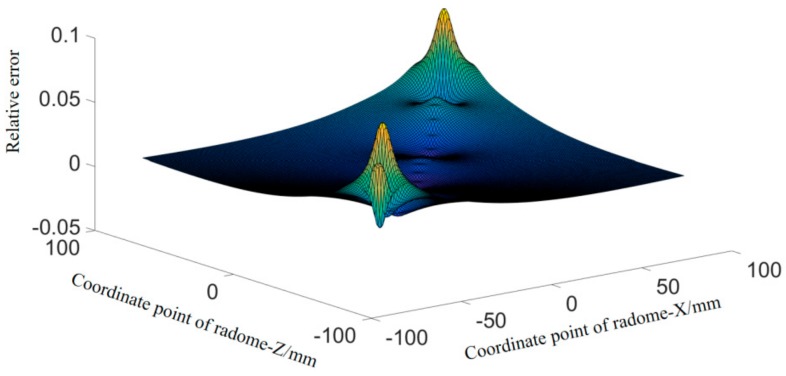
Accuracy error “cloud map”.

The result is shown in Figure 5.

By using the least-squares method for the glass radome complex surface fitting, the following conclusions could be drawn: the glass radome complex surface function can be expressed by the polynomial expression of the introduction or the more complicated Fourier expansion form, both of which have a better overall fitting effect. However, in addition to ensuring the overall fitting effect, the Fourier expanded form expression effectively avoids the bad point, so it is recommended that the Fourier expansion form is used for the fitting function of the complex surface of the glass radome.

### 2.2. Trajectory Planning for Abrasive Belt Grinding

Through the least-squares fitting of the projection curve of the outer surface of the FRP radome, the prediction equation of the FRP smooth surface radome was obtained. However, in the actual abrasive belt grinding process, the abrasive belt is in surface contact with the grinding member rather than in point contact, and its display on the FRP projection surface is the line contact. For this reason, when planning the path of the belt, the projection curve should be evenly decomposed into several sections to describe the actual situation of sanding.

For the FRP radome, the general grinding force was about 10–15 N, and the corresponding projection grinding line was about 5–6 mm. The fitting curve length was calculated by integrating the fitting curve by the least-squares Fourier approximation. For the microelements, $ds=\frac{dx}{cos\theta }$ and $tan\theta =f^{\prime }\left(x\right)$(*f(x*) is the fitted curve equation, from which

(9) $$\begin{array}{c} ds=\sqrt{1+{\left[f^{\prime }\left(x\right)\right]}^{2}}dx \end{array}$$

can be obtained, so

(10) $$\begin{array}{c} s=\int_{a}^{b}\sqrt{1+{\left[f^{\prime }\left(x\right)\right]}^{2}}dx.\; \end{array}$$

The calculated length of the FRP radome arc was 194 mm, which was about 38 times that of the surface contact. For the machining done on a belt grinding CNC (Computer Numerical Control) machine, the specific point coordinates were input, and then the machine automatically fit the trajectory curve. According to the contact characteristics of the abrasive belt grinding surface, for the FRP radome, each contact can find a corresponding point, so the curve of the FRP radome only needed to find 38 points as its target corresponding point (*A*, *B* are the start and end points of the curve, respectively).

**Figure 6:**
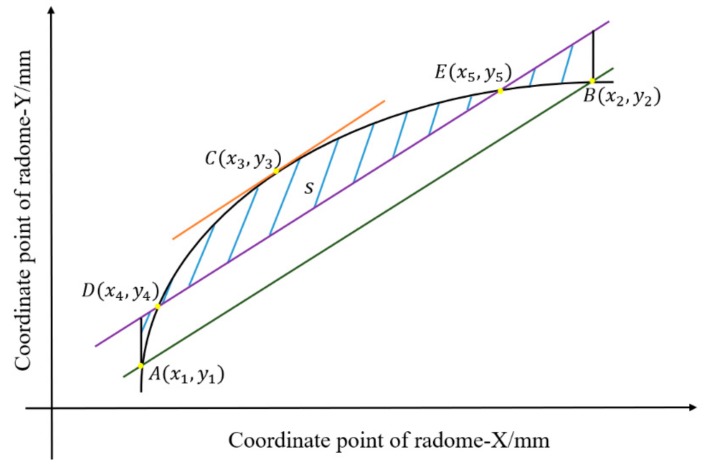
Corresponding point determination diagram.

The method used for determining the selection of one of the points was shown in Figure 6:

Take any one of the 39 straight lines, the fitting curve of which is evenly divided into lengths, namely,

(11) $$\begin{array}{c} \hat{AB}=Belt\;unit\;line. \end{array}$$

Connect *AB* with a slope of $k=\frac{y_{2}-y_{1}}{x_{2}-x_{1}}$. Take a point and make its partial derivative *k*, which is the target coordinate point *C*. The coordinates of the target $C(x_{3},y_{3})$ are obtained by the following equations:

(12) $$\begin{array}{c} \begin{array}{c} \left\{\begin{array}{c} y_{3}=f\left(x_{3}\right)\\ f^{\prime }\left(x_{3}\right)=\frac{y_{2}-y_{1}}{x_{2}-x_{1}} \end{array}\right. \end{array}\\ \left(f\left(x\right)is\;the\;equation\;for\;fitting\;the\;curve\right). \end{array}$$

Then, make a straight line with a slope of $f^{\prime }\left(x_{3}\right)$ to fit the curve to two points, $D\left(x_{4},y_{4}\right)$ and $E\left(x_{5},y_{5}\right)$, which fulfill the requirement that the area S is the smallest. The line segment $\hat{DE}$ is the trajectory path of the abrasive belt, and S depicts the error between the abrasive belt grinding track and the fitting prediction curve.

The coordinates obtained by this method have the characteristics of universality and high precision considering the contact characteristics of the grinding surface.

To this end, 38 target points were obtained by the above method, as shown in Table 2.

**Figure 7:**
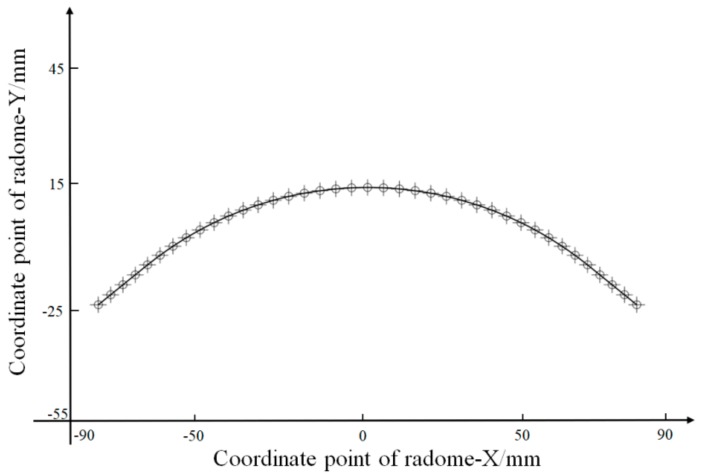
Curve coordinate point line uniformity division diagram.

**Figure 8:**
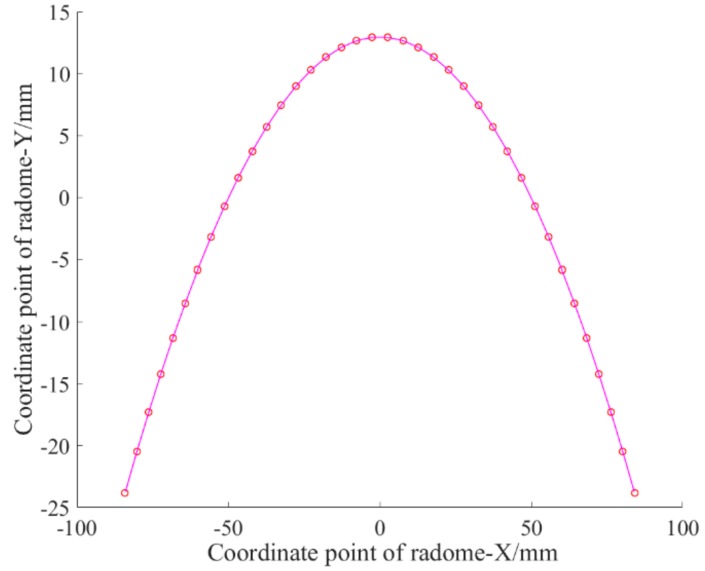
Curve line interpolation map.

According to the data, the curve was evenly divided, as shown in Figure 7, and the linear interpolation based on these points yielded the results shown in Figure 8.

**Figure 9:**
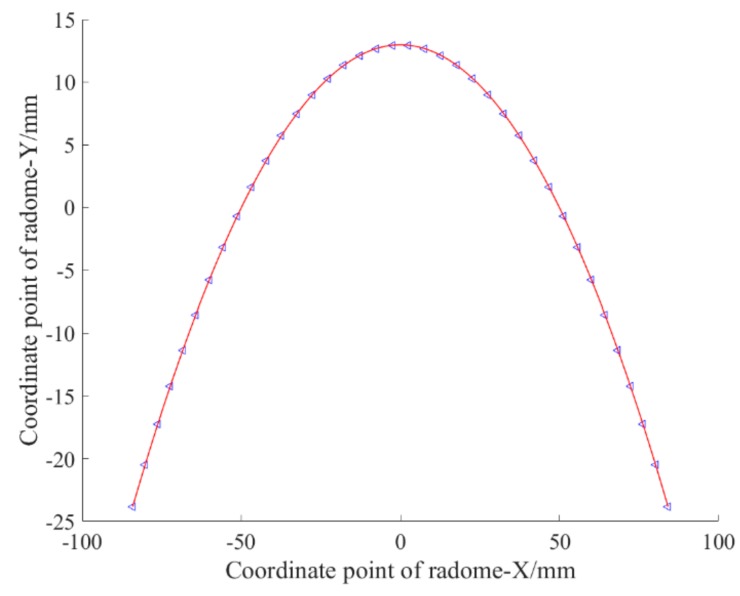
Fourier form fitting curve for the target point.

Fourier approximation was used to fit the curve and the result was in Figure 9:

$$\begin{array}{c} f\left(x\right)=-388.8+426.6\cos\left(\mathrm{xw}\right)-73.41\cos\left(2\mathrm{xw}\right)+14.88\cos\left(3\mathrm{xw}\right)-2.603\cos\left(4\mathrm{wx}\right)\\ +0.3174\cos\left(5\mathrm{wx}\right)-0.01946\cos\left(6\mathrm{wx}\right)w=0.01164. \end{array}$$

The fitting effect was *SSE* = 9.808 × 10^−21^, *R*^2^ = 1, and C = 0.02%. The overall fitting effect was better.

**Figure 10:**
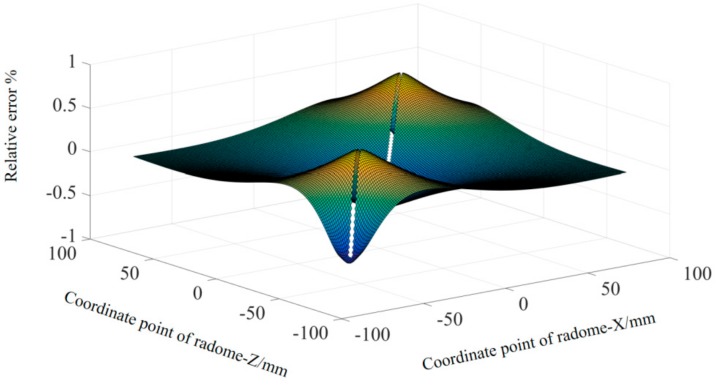
Accuracy error cloud map.

To this end, the obtained abrasive belt grinding trajectory curve equation and the fitted ideal radome curve equation were used for error analysis. The accuracy error cloud diagram is shown in Figure 10.

The accuracy error cloud diagram (Figure 10) revealed that the relative error between the abrasive belt trajectory curve equation and the ideal radome curve equation was within 1%. That is, the trajectory generation under this method was more effective.

## 3. Materials and Methods

### 3.1. Materials

**Figure 11:**
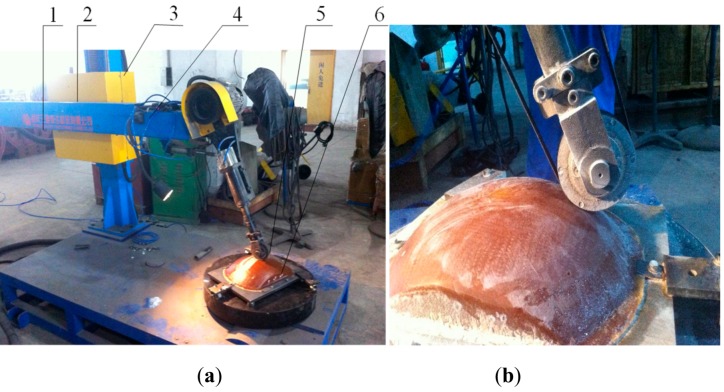
Experimental machine diagram. (**a**)—Grinding overall diagram. (**b**)—Grinding partial close-up diagram. 1—Cross host. 2—Cross slide. 3—Column. 4—Beam. 5—Standard grinding head. 6—Antenna cover.

The machine tool used in the experiment was the industrial tank body polishing machine 2M58200DE (Chongqing Sanmo Haida Grinding Machine Co., Ltd., Chongqing, China), which adopted a numerical control three-axis machining method and was specially used for grinding the inner and outer surfaces of the cylinders of large- and medium-sized industrial tanks. The grinding of the complex curved surface parts, such as the rotary and head surfaces, can be controlled by a high-grade CNC system to roughen, finish, and polish the surface of a work piece. In addition to being able to machine the surface well, the machine can also perform the polishing process on the edge of the surface, avoiding the inefficiency of complex surface processing, poor surface quality, and poor product consistency. The machine tool used here included a cross host, cross slide, column, beam standard grinding head, and antenna cover, as shown in Figure 11. Table 3 shows the main technical parameters of the selected machine tool.

**Figure 12:**
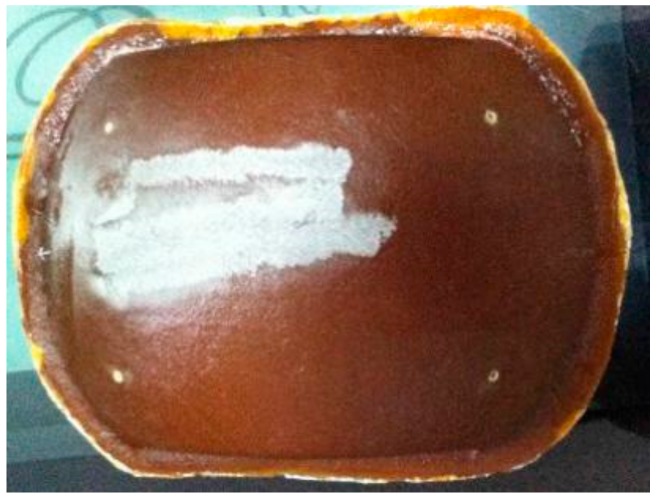
Unground radome diagram.

**Figure 13:**
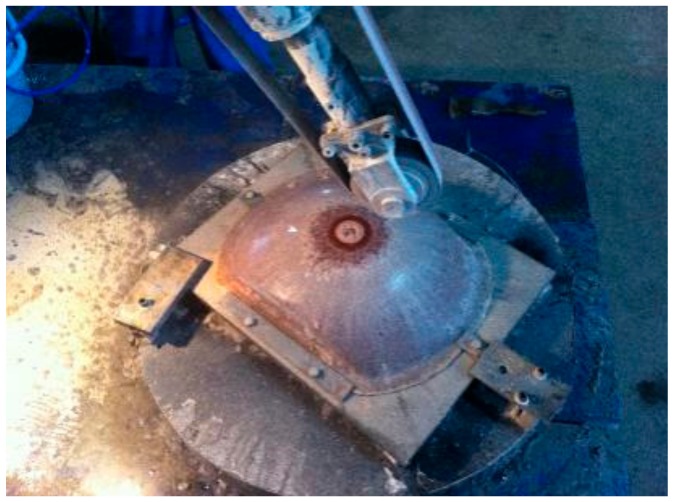
Radome in grinding.

The ungrounded work piece used in the experiment is shown in Figure 12. It was a roughened parabolic radome with a density of 2.06 g/cm^3^ and a surface roughness (Ra) of 1.2–1.8 μm with an average volume of 1.56 μm. The radome during grinding is shown in Figure 13.

The ungrounded work piece used in the experiment is shown in Figure 12. It was a roughened parabolic radome—an FRP composite with a density of about 2.06 g/cm^3^ and an Ra of 1.3–1.8 μm (the average was 1.56 μm). The radome during the grinding process is shown in Figure 13. 

### 3.2. Methods

Before the work, the FRP radome was ground and the work piece was prepared to ensure the surface the neatness of the work piece. The experiment used a ceramic abrasive belt with a size of P120. The specification of the belt was 10 × 2540 mm (width × circumference). The wet grinding method was adopted, the belt was driven by constant turbulence, and the processing route was also processed by row cutting.

According to the results of the previous experiments, the process parameters of ceramic abrasive grinding were a spindle speed n = 1800 r/min and a feed rate v = 2800 mm/min.

**Figure 14:**
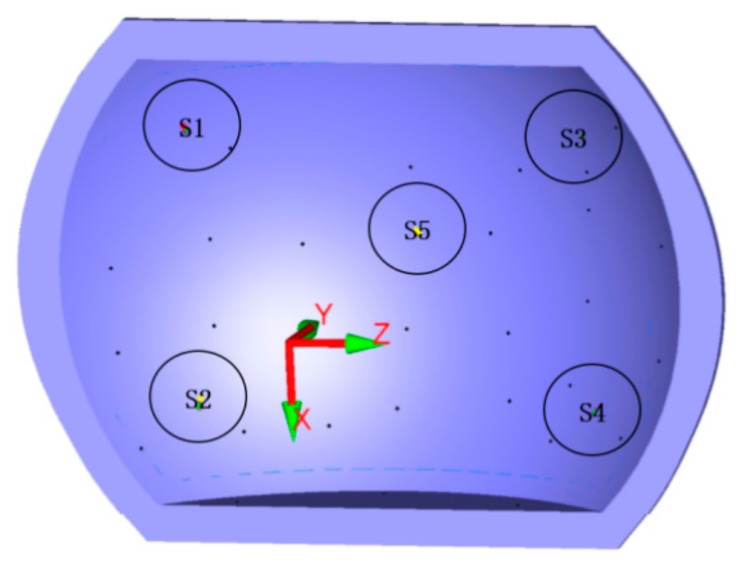
Target points in five areas on the grinding surface.

In order to fully reflect the difference in the contour accuracy of the entire grinding process under different algorithms, the grinding surface was divided into five regions. According to the surface features, the five regions were selected from the vertices and edges of the surface, including one vertex and four peripherals, as shown in Figure 14.

For the roughness measurement and contour accuracy analysis, each area was measured five times, then the maximum and minimum values were removed, and the remaining three datasets were averaged to reduce the measurement error and improve the accuracy. The measurement of the three-dimensional shape was not particularly complicated, and it was only necessary to detect and record the three-dimensional shape of each position.

## 4. Results

### 4.1. Contour Accuracy Analysis

For the accuracy analysis of the contour, the conventional grinding path setting and the contour error of the grinding path generated using the trajectory optimization algorithm were compared.

The error accuracy of each point was characterized by *DEV* (relative deviation/mm). It is expressed as the relative deviation caused by grinding under the traditional algorithm and the relative deviation caused by grinding under the trajectory optimization algorithm. Among them are indicators that characterize the overall relative deviation:

(13) $$\begin{array}{c} DEV_{s1,2}=\sqrt{DEV_{x1,2}{}^{2}+DEV_{y1,2}{}^{2}+DEV_{z1,2}{}^{2}}. \end{array}$$

(14) $$\begin{array}{c} R_{i}=\frac{DEV_{i1}-DEV_{i2}}{DEV_{i1}}\left(i=x,y,z,s\right),\; \end{array}$$

where $R_{i}$ characterizes the relative deviation of the trajectory optimization algorithm compared to the traditional algorithm.

**Figure 15:**
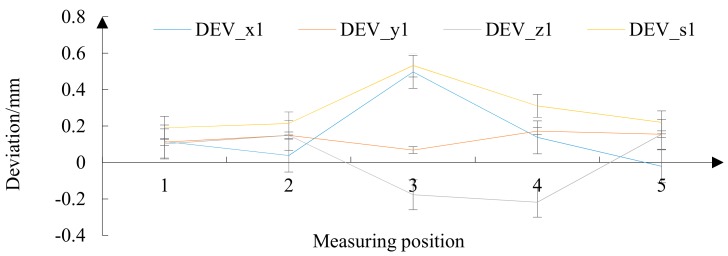
Relative deviation under traditional algorithm.

From the relative deviation of the traditional algorithm shown in Figure 15, it can be seen that the accuracy error fluctuations in the three directions were larger, and the error peak was higher. Among them, the overall relative deviation $DEV_{s1}$ had a maximum of 0.532 mm and a minimum of 0.19 mm. The surface precision error of the FRP radome under grinding by the traditional algorithm was large and the variance was not uniform.

**Figure 16:**
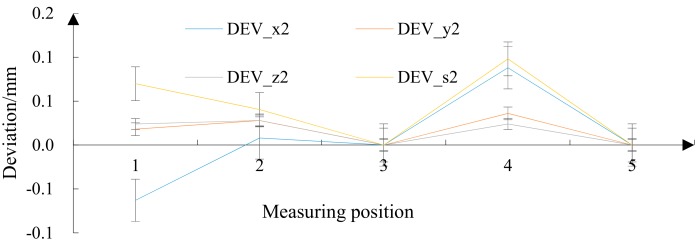
Relative deviation under the trajectory optimization algorithm.

From the relative deviation of the trajectory optimization algorithm (Figure 16), it can be seen that the accuracy error fluctuations in the three directions were relatively small, and the error peak was more diversified. The overall relative deviation ${\mathrm{DEV}}_{s2}$ of 0.098 mm was the highest in the five regions and the lowest was 0 mm. Under the trajectory optimization algorithm, the surface precision error of the FRP radome was small, and the variance was relatively small.

**Figure 17:**
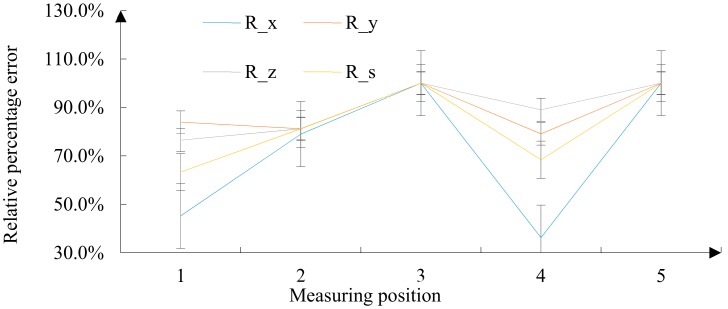
Relative deviation of the traditional algorithm compared to the trajectory optimization algorithm.

From the relative deviation of the traditional algorithm compared to the trajectory optimization algorithm (Figure 17), the precision error caused by grinding under the traditional algorithm was the main reason for the accuracy error.

The merits and demerits of the two calculations were evaluated by the root-mean-square error (RMSE):

(15) $$\begin{array}{c} RMSE=\sqrt{\frac{\sum_{m=1}^{n}{\left(I_{obs,m}-I_{model,m}\right)}^{2}}{n}}\left(I=x,y,z\right) \end{array}$$

$$I_{obs,m}:\;the\;scale\;measurement\;in\;a\;certain\;direction\;after\;grinding$$

$$I_{model,m}:\;\mathrm{the}\;\mathrm{theoretical}\;\mathrm{value}\;\mathrm{of}\;\mathrm{the}\;\mathrm{scale}\;\mathrm{in}\;a\;\mathrm{certain}\;\mathrm{direction}.$$

After calculation, $RMSE_{traditiomal}=0.3681$ and $RMSE_{thenew}=0.044$. The accuracy of the trajectory optimization algorithm was roughly 16.8% higher than that of the conventional algorithm.

### 4.2. Surface Integrity

**Figure 18:**
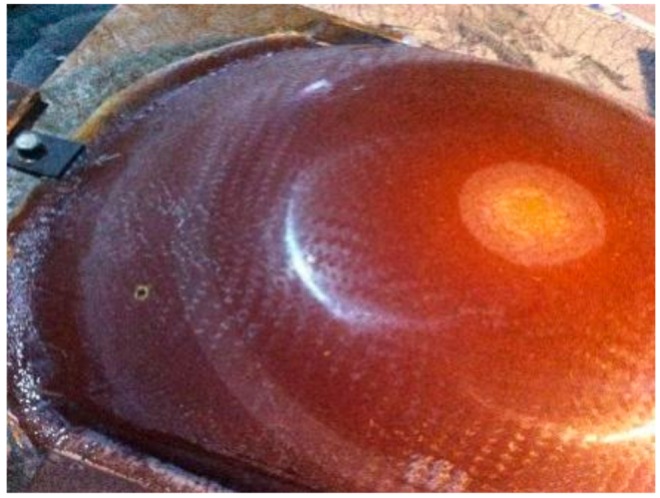
Grinding radome.

Figure 18 shows the physical diagram of the radome after the grinding process was completed. In order to compare the advantages and disadvantages of the traditional algorithm and the trajectory optimization algorithm, surface roughness and three-dimensional-shape analyses of the physical object were respectively performed.

**Figure 19:**
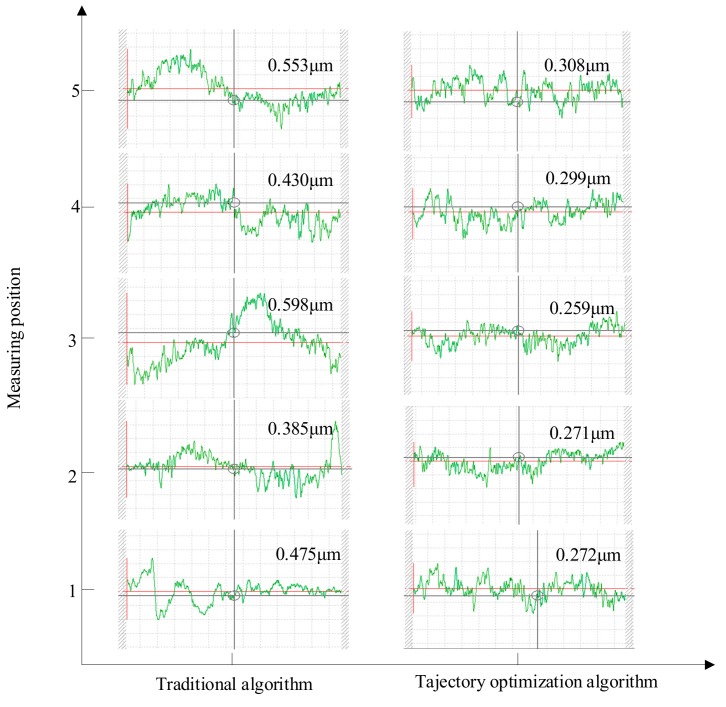
Roughness comparison in five regions under the two algorithms.

The above selection of the five areas on the grinding surface was still used. The roughness of each area was measured and is shown in Figure 19.

It can be seen from Figure 19 that the amplitude of the grinding surface roughness fluctuation under the conventional algorithm was large, the overall roughness value was large, the extreme difference between the upper and lower limits was large, the maximum range difference was 0.4 μm, and the surface consistency was poor.

The surface roughness of the grinding measurement point under the trajectory optimization algorithm was very stable. Except for the small roughness (Ra) of the whole object, the roughness of the region where the measurement point after grinding was smaller was 0.271 μm. The minimum difference of the lower limit was also very small, and the surface consistency was good.

**Figure 20:**
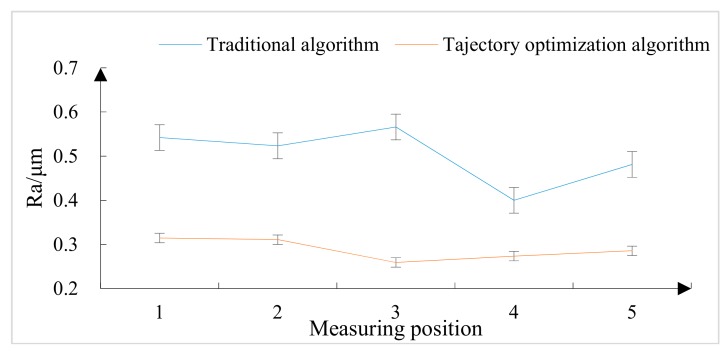
Traditional algorithm and trajectory optimization algorithm experimental roughness measurement line diagram.

From the distribution law of the roughness of the measurement points and the roughness of the measurement points, the roughness of the trajectory optimization algorithm was lower than the roughness of the conventional algorithm as a whole as shown in Figure 20. The roughness in the five regions under the conventional algorithm had a maximum roughness of 0.566 μm and a minimum value of 0.400 μm. The roughness of the five regions of the trajectory optimization algorithm had a maximum roughness of 0.315 μm and a minimum roughness of 0.260 μm. For the overall quality, the grinding surface roughness under the conventional algorithm was 0.503 μm, and the grinding surface roughness under the trajectory optimization algorithm was 0.289 μm. In terms of overall roughness level, the trajectory optimization algorithm was far superior to the traditional algorithm.

**Figure 21:**
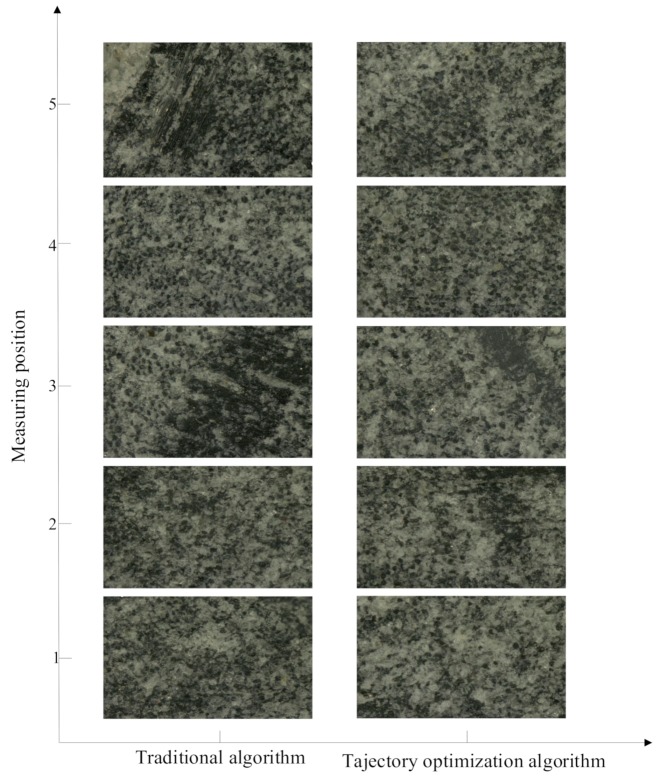
The topographical features when the magnification was 100×.

**Figure 22:**
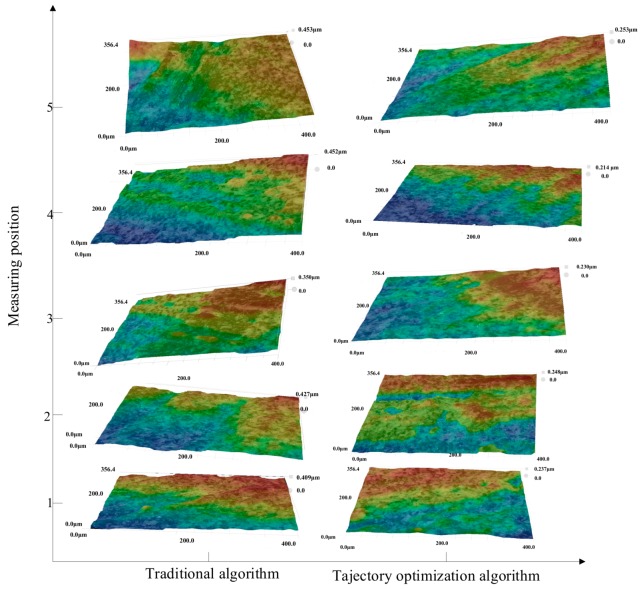
Superdepth three-dimensional topography of the five regions under two algorithms.

Figure 21 shows the topographical feature map when the magnification was 100×, and Figure 22 shows the three-dimensional topographical map of the superdepth of field in the five regions.

It can be seen in Figure 21 that the distribution of the microscopic crystals in the conventional algorithm was not uniform but distributed towards a certain grinding trace, and the grinding texture can be clearly observed, as well as the crystal in the low-light shape under the trajectory optimization algorithm. The particle distribution was relatively uniform.

It can be seen from the three-dimensional topographical map of the five regions in Figure 22 that the five-region three-dimensional topographic maps under the traditional algorithm were 0.409, 0.427, 0.350, 0.452, and 0.453 μm, respectively. The peak heights of the three-dimensional topographic maps under the trajectory optimization algorithm were 0.237, 0.248, 0.230, 0.214, and 0.253 μm, respectively.

In summary, the surface integrity of the trajectory optimization algorithm after grinding was better than the surface integrity after grinding by the traditional algorithm.

## 5. Conclusions

Mathematical experiments were conducted on the complex curved surface of a glass radome. Considering the full symmetry of the complex surface of the glass radome, the curve fitting of the projection surface of the complex surface was carried out using the least-squares method employing polynomial approximation, least-squares optimization fitting, and Fourier approximation. Among them, the Fourier approximation effect was optimal, and its fitting effect was *SSE* = 0.0098, *R*^2^ = 1.000, and C = 0.6%. For this reason, the Fourier approximation form was adopted for the fitting of the complex curve, and the three-dimensional precision error cloud map was obtained by the Fourier approximation function. It can be seen that the overall fitting effect was excellent. To this end, the surface fitted by the Fourier approximation was used as the ideal function surface of the radome.

Considering the characteristics of the belt surface contact, when planning the belt path of the belt, the projection curve was evenly decomposed into several sections, and the surface contact point of each section was found on the ideal curve by the surface contact grinding characteristic as a sample point for its trajectory planning. Then, based on the Fourier approximation fitting of the least-squares method, the path of the abrasive belt grinding path was obtained by fitting. The fitting effect was $\mathrm{SSE}=\;9.808\times 10^{-21}$, *R*^2^ = 1, and C = 0.2%. The three-dimensional precision error cloud map of the curve with the ideal was made, and the maximum relative deviation was within 1%. The trajectory generation under this method was shown to be more effective.

For the analysis of the experimental results, in order to compare the advantages and disadvantages of the traditional algorithm and the trajectory optimization algorithm, contour accuracy and surface integrity analyses were used. For the contour accuracy analysis, the grinding surface was divided into five regions, the error accuracy of each point was characterized by DEV (relative deviation/mm), and the precision errors of grinding the surface under the two different algorithms were compared. 

The precision error under the traditional algorithm fluctuated greatly and the peak value was high. Compared with the trajectory optimization algorithm, the error precision was small and stable. The accuracy of the trajectory optimization algorithm was roughly 16.8% higher than that of the conventional algorithm. 

For the analysis of surface integrity, surface roughness and three-dimensional-shape analyses of the physical object were carried out. The selection of the five regions on the grinding surface was still adopted. The roughness of the contrast trajectory optimization algorithm was generally better than the traditional algorithm, and the roughness was lower. Moreover, the surface roughness of the grinding measurement point under the trajectory optimization algorithm was very stable, the peak height of the superdepth of field three-dimensional macromap was relatively low, and the distribution of crystal particles under the low-light shape was relatively uniform. The comparison of the average surface roughness results under the different algorithms is shown in Table 4.

For these reasons, the surface integrity after grinding using the trajectory optimization algorithm is considered to be superior to the surface integrity after grinding by the traditional algorithm.

## Figures and Tables

**Table 1:** Relative coordinates of sample points under digital-to-analog conversion.

Number	Relative Position X	Relative Position Y	Number	Relative Position X	Relative Position Y
**1**	82.943	−23.279	**24**	−3.8173	12.385
**2**	79.795	−20.676	**25**	−7.6255	12.187
**3**	76.646	−18.073	**26**	−11.424	11.858
**4**	73.498	−15.469	**27**	−15.21	11.398
**5**	67.369	−10.487	**28**	−18.977	10.807
**6**	64.308	−8.2131	**29**	−22.722	10.087
**7**	61.171	−6.0457	**30**	−26.439	9.2382
**8**	57.961	−3.9879	**31**	−30.125	8.2612
**9**	54.681	−2.0422	**32**	−33.775	7.1574
**10**	51.336	−0.211	**33**	−37.385	5.9282
**11**	47.93	1.5037	**34**	−40.95	4.5751
**12**	44.467	3.0997	**35**	−44.467	3.0997
**13**	40.95	4.5751	**36**	−47.93	1.5037
**14**	37.385	5.9282	**37**	−51.336	−0.211
**15**	33.775	7.1574	**38**	−54.681	−2.0422
**16**	30.125	8.2612	**39**	−57.96	−3.9879
**17**	26.439	9.2382	**40**	−61.171	−6.0457
**18**	22.722	10.087	**41**	−64.308	−8.2131
**19**	18.977	10.807	**42**	−67.369	−10.487
**20**	15.21	11.398	**43**	−73.498	−15.469
**21**	11.424	11.852	**44**	−76.646	−18.073
**22**	7.6256	12.187	**45**	−79.795	−20.676
**23**	3.8174	12.385	**46**	−82.943	−23.279

**Table 2:** Abrasive belt grinding track target points.

Number	Relative Position X	Relative Position Y	Number	Relative Position X	Relative Position Y
**1**	−84.123	−23.829	**20**	−3.817	12.385
**2**	−80.188	−20.466	**21**	−7.625	12.187
**3**	−76.252	−17.265	**22**	−11.424	11.858
**4**	−72.317	−14.225	**23**	−15.213	11.398
**5**	−68.353	−11.325	**24**	−18.977	10.807
**6**	−64.291	−8.523	**25**	−22.722	10.087
**7**	−60.093	−5.808	**26**	−26.439	9.238
**8**	−55.767	−3.201	**27**	−30.125	8.261
**9**	−51.322	−0.726	**28**	−33.775	7.157
**10**	−46.769	1.595	**29**	−37.385	5.928
**11**	−42.117	3.746	**30**	−40.953	4.575
**12**	−37.375	5.706	**31**	−44.467	3.099
**13**	−32.554	7.459	**32**	−47.935	1.503
**14**	−27.664	8.990	**33**	−51.336	−0.211
**15**	−22.715	10.286	**34**	−54.681	−2.042
**16**	−17.719	11.337	**35**	−57.962	−3.987
**17**	−12.684	12.133	**36**	−61.171	−6.045
**18**	−7.623	12.667	**37**	−64.308	−8.213
**19**	−2.545	12.936	**38**	−67.369	−10.487

**Table 3:** Main technical parameters of machine tools.

Parameter	Range
Turntable diameter (mm)	1500
Turntable speed (r/min) frequency conversion adjustable	1–20
Processing barrel and head diameter (mm)	Φ800–Φ4000
Throwing efficiency ($m^{2}$/h)	6–10
Surface roughness, Ra (μm)	0.1–0.4
Contact wheel size (mm)	Φ25–Φ180
*x*/*y*/*z*-axis repeat positioning accuracy	≤0.01 mm

**Table 4:** Comparison of average surface roughness results under the different algorithms.

	Surface Average Roughness
**Traditional algorithm**	0.503 μm
**Trajectory optimization algorithm**	0.289 μm
